# Biometrics and Biosecurity 2013

**DOI:** 10.1155/2013/734642

**Published:** 2013-12-10

**Authors:** Tai-hoon Kim, Sabah Mohammed, Wai-Chi Fang

**Affiliations:** ^1^Department of Convergence Security, Sungshin Women's University, Dongseon-dong-3-ga, Seongbuk-gu, Seoul, Republic of Korea; ^2^Department of Computer Science, Lakehead University, 955 Oliver Road, Thunder Bay, ON, Canada P7B 5E1; ^3^National Chiao Tung University, 1001 Ta Hsueh Road, Hsinchu 300, Taiwan

We are very happy to publish this special issue. This issue contains 11 articles that come from various countries, among which we mention Saudi Arabia, Republic of Korea, Macau, Canada, Australia, China, the Czech Republic, India, and Japan.

Biometrics and Biosecurity 2013 focused on the various aspects of advances in biometrics and biosecurity. This special issue will provide a chance for academic and industry professionals to discuss recent progress, problems, and solutions in the area of biometrics and its application, biosecurity measures, and biosafety protocols, including development, implementation, strategies, and policies.

In “*An improved biometrics-based remote user authentication scheme with user anonymity*,” the authors proposed an improved scheme eradicating the flaws of the previous scheme. The proposed scheme not only withstands security problems found in the previous scheme but also provides some extra features with the mere addition of only two hash operations.

In the paper “*The quantitative overhead analysis for effective task migration in biosensor networks*,” authors presented a quantitative overhead analysis for effective task migration in biosensor networks. A biosensor network is the key technology which can automatically provide accurate and specific parameters of a human in real time. The results of performance evaluation showed that task execution time is greatly influenced by a cluster ratio and different processing time of biosensor nodes.

The objective of the paper “*Evaluation of stream mining classifiers for real-time clinical decision support system: a case study of blood glucose prediction in diabetes therapy*” is to find out the most suitable classifier for rt-CDSS, and therefore the authors compared them in a diabetes therapy scenario. Also the authors tested the performance of the classifier candidate all-rounded with a real-time case study, as a preliminary step to validate the efficacy of the rt-CDSS as a whole.

In “*Classifying human voices by using hybrid SFX time-series preprocessing and ensemble feature selection*,” the authors focused on the comparison of effects of various popular data mining algorithms on multiple datasets. The authors' experiment consisted of classification tests over four typical categories of human voice data, namely, female and male, emotional speech, speaker identification, and language recognition.

In the paper “*Secure encapsulation and publication of biological services in the cloud computing environment*,” secure encapsulation and publication for bioinformatics software products based on web service were presented and the basic function of biological information was realized in the cloud computing environment. In the encapsulation phase, the workflow and function of the bioinformatics software were conducted, the encapsulation interfaces were designed, and the runtime interaction between users and computers was simulated. In the publication phase, the execution and management mechanisms and principles of the GRAM components were analyzed.

In “*Image analysis of endoscopic ultrasonography in submucosal tumor using fuzzy inference*,” the authors proposed a method to extract areas of GIST and lipoma automatically from the standardized ultrasonic image to assist those endoscopists. The authors also proposed an algorithm to differentiate GIST from non-GIST by fuzzy inference from such images after applying an ROC curve with mean and standard deviation of the brightness information.

In “*A study on user authentication methodology using numeric password and fingerprint biometric information*,” user authentication was performed that uses biometric information and passwords of users. The user cannot change user's fingerprint information, but the user has set a password to change easily. So this authentication system provides security and flexibility. Because it makes a password key that utilizes the user's fingerprint and numeric password, an attacker does not take advantage of leaked passwords.

This article “*New optical methods for liveness detection on fingers*” was devoted to new optical methods, which are supposed to be used for liveness detection on fingers. First the authors described basics about fake finger use in the fingerprint recognition process and possibilities of liveness detection. Then the authors continued with introduction of three new liveness detection methods, which the authors developed and tested in the scope of the authors' research activities—the first one was based on measurement of pulse, the second one was based on variations of optical characteristics caused by pressure change, and the last one was based on reaction of skin to illumination with different wavelengths.

In the paper “*Designing a bioEngine for detection and analysis of base string on an affected sequence in high-concentration regions*,” authors designed an algorithm for the bioengine. Searching for homologues had become a routine operation of biological sequences in 4 × 4 combination with a different subsequence (word size). This program takes advantage of the high degree of homology between such sequences to construct an alignment of the matching regions.

In “*Statistical fractal models based on GND-PCA and its application on classification of liver diseases*,” a new method was proposed to establish the statistical fractal model for liver diseases classification. Firstly, the fractal theory was used to construct the high-order tensor, and then Generalized N-dimensional principal component analysis (GND-PCA) was used to establish the statistical fractal model and select the feature from the region of the liver; at the same time different features had different weights. Finally, the support vector machine optimized ant colony (ACO-SVM) algorithm was used to establish the classifier for the recognition of the liver disease.

The paper “*HyDEn: a hybrid steganocryptographic approach for data encryption using randomized error-correcting DNA codes*” presented a novel hybrid DNA Encryption (HyDEn) approach that uses randomized assignments of unique error-correcting DNA Hamming code words for single characters in the extended ASCII set. HyDEn relied on custom-built quaternary codes and a private key used in the randomized assignment of code words and the cyclic permutations applied to the encoded message.

